# Mr. Hume on the Detection of Arsenic

**Published:** 1818-06

**Authors:** 


*For the London Medical and Physical Journal.*

### Mr. Hume *on the Detection of Arsenic.*

BEFORE the completion of the 39th volume of your valuable Journal, I wish to testify to your correspondents, Messrs. Kerr and Prideaux, that I have read their remarks on my paper, on the detection of arsenic, and, I trust, with my best attention. I confess, however, that I could find nothing new in the form of reasoning, theory, or experiment to invalidate the plans, taken collectively, which I have advised for discovering this poison. I say taken *collectively*, for I have repeatedly insisted on the advantage of tests supporting and confirming each other,—forming reciprocal proofs; and thus, in the most conclusive way, rendering the evidence incontrovertible.

I hope to hear no more arguments upon that preposterous story of the decoction of onions. If Mr. Kerr will still maintain his opinion on this subject, it will, I fear, be in vain for me to attempt a refutation, even by experiment, —for that is the only way. I here positively deny that this vegetable can influence the different re-agents. Dr. Plenderleath, Mr. Marshall, and others, have made the experiments, and these, as well as some of my own, are before the public.

Mr. Kerr should give some reason for omitting the *carbonate of potash* in the two experiments, or rather contrivances, detailed at page 93 of this volume. I call them contrivances, because they are avowedly meant to imitate what Dr. Edwards is supposed to have found in the contents of the stomach, obtained *post mortem*, viz. phosphate of soda, and decoction of onions! Nothing can be more frivolous than the reasoning and inferences drawn from this history. It is well known, that the most ignorant cook strains off and rejects the decoction, retaining only the vegetable, exhausted of the very principles with which Dr. Neale and others made their experiments. Had Mr. Kerr used the carbonate (probably *subcarbonate*,) of potash, would he have had the same results? Certainly not; there could be no refutation of Dr. Edwards's opinion displayed by such management. To believe that any decoction of onions could possibly remain after so much food and medicine had been taken,—after so many hours had elapsed, and after such violent retchings, evacuations, and convulsive disturbance of the animal frame, requires more faith than I possess.

Mr. Prideaux puts a forced construction on my language, to suppose that any fellow-creature should be condemned upon no other evidence than swearing to the presence of arsenic merely by *one* experiment. I should myself be most scrupulous in giving my testimony, and even backward in my exertions, where the collateral, presumptive, and circumstantial proofs were deficient or very slight: were these, however, to be conspicuous and well supported, I would apply my best abilities to obtain the truth and convict the guilty. I beg to refer this gentleman to my last paper, in your Journal for January, where I have said, that "should I fail on any *interesting* occasion, let my evidence, as all other doubtful testimony should, go to the *merciful* scale of the balance;" that is, in case I should fail to satisfy my own conscience and professional conviction.

I am not inclined, nor will my time allow me, to enter into a more extended comment upon Mr. Kerr's last letter, which, as well as that of Mr. Prideaux, may probably be more fully noticed in one of your future numbers. I cannot admit Mr. Prideaux's assertion, that "phosphate of soda does assuredly exist in the gastric fluid." This gentleman often speaks so hypothetically, that I am at some loss how to reply to many parts of his letter. Presuming that the phosphate of soda exists, "quantity *may* not be such, in *some* states of disease, &c." "*Supposing* the person to die of cholera morbus,"—"It *may be doubted*, &c." "*May* not some oxides or subsalts," &c. Mr. Prideaux observes, that "when the alkali is applied *first*, we cannot be deceived." Now, the alkali is in some cases quite superfluous; it is altogether out of its place where phosphates are, and equally so should Fowler's solution or combined arsenic exist. An additional alkali is improper when *ammoniaco*-nitrate of silver is to be applied, and this form of applying the re-agent has not been duly estimated by Mr. Prideaux. If *simple* nitrate of silver causes a yellow precipitate in any fluid, it forms a case of ambiguity; if it does not produce such effect, until the moistened paper with ammonia* be held over the surface, there can be no doubt of the presence of arsenic, provided the solution be free from *uncombined acid.* There are many methods for getting rid of the phosphates, prior to our search for the arsenic. I shall not here repeat what I have often mentioned, respecting *lime* being incompatible with soluble phosphates, and that these may be readily decomposed by its application. Mr. Prideaux's objection to ammoniaco-nitrate of silver, that it "is not applicable, as it would entangle the arsenic in so overwhelming a quantity of muriate, as to be hardly separable by any means," is what I do not fully understand. *Simple* nitrate of silver will separate all the muriatic acid, and leave the arsenic in solution: and, supposing the solution to contain nothing but muriate of soda and white arsenic, the ammoniaco-nitrate would then detect the arsenic.

But to be brief, which is my present intention,—give me the *yellow* precipitate by means of silver, whether a phosphate or an arsenite, or let them be mixed, and I am very confident, the arsenic may, under all these difficulties, be extricated, and shewn in the state of *white oxide* or arsenious acid, which is by far the most certain mode of collecting it for the purpose of evidence. In this condition, when fairly elicited in a glass vessel of appropriate shape and dimensions, the same quantity of the poison will occupy more space, be more palpable and tangible than metallic arsenic, and can be, in the most convincing way, once more identified by the ammoniaco-nitrate of silver. To conclude, let me ask Messrs. Kerr and Prideaux this question:—Suppose a little of the phosphate of soda, muriate of soda, and white oxide of arsenic, were mixed and exposed in a glass vessel to the heat of a spirit-lamp, would the arsenic leave the salts, and attach itself to the superior parts of the vessel?

To Mr. Kerr's question, page 191, I most willingly answer, by informing him, that, profiting by the errors of others, I formed the triple salt, *ammoniaco*-nitrate of silver, entirely with a view to precision, and to prevent any excess of alkali, where the arsenic is supposed or found to be uncombined. It was not till very lately that I perceived the singular utility of this modification,—that of not affecting the *phosphate of soda*, which, so far, is a most valuable occurrence: I am not prepared, however, to say that it refuses to act with the other soluble phosphates.

---

* See my last paper,—Journal for January, Page 27.

I cannot concur with Mr. Prideaux, who contends for the proof of metallic reduction as the *only* evidence that ought to be admitted. Who that is but moderately conversant in practical science, would think of reducing "a quarter of a grain, and, *probably*, a much smaller quantity," into the metallic form, when the truth can be so much more easily and forcibly substantiated *viâ humidâ*. I agree perfectly with this gentleman, that "a novice in chemistry might, without unjust design, occasion the death of an innocent person, by want of skill in investigation." But is not this a two-edged sword; is there not an equal chance that the same ignorant and blundering novice may prevent conviction and its consequent just punishment? Does not the same objection hold against every kind of technical evidence? Indeed a case in point has just come to my knowledge, and, as Mr. Prideaux's name and character are not implicated, I do not scruple to mention it here. A collection of minerals at Plymouth was shewn to a friend of mine; and, amongst other specimens, the possessor shewed what he considered as a very curious substance: it was labelled, calcareous spar, or *carbonate of lime*,—and, although now quite opaque and white, was described as having been originally perfectly translucid, and similar to glass. Now this *rara avis* was instantly recognized by the visitor to be neither more nor less than *white oxide of Arsenic!* Supposing this collector of minerals, or geological specimens, were called upon to give his evidence, what faith could be placed in his *practical* acquirements and *technical* knowledge? I am unwilling to press this anecdote farther, otherwise I might be led to make some pointed reflections on a subject that has been forced upon me by the officiousness of others.

I shall now beg leave to assure both Mr. Kerr and Mr. Prideaux, that I take every thing they have said in good part, believing they are actuated by the best of motives,—benefit to society, and security in the due administration of public justice: in this road I am ready at all times, when other avocations will allow me, to go hand in hand over the fields of enquiry, and this communication shall be an earnest of my good intentions. Let us, however, scrupulously refrain from further remarks on decoction of onions, the experiments, and all the *nugæ canoræ* detailed as evidence on a late trial; for, on that subject, my mind is fixed, and my opinion remains inflexible.

*Long Acre, May* 14, 1818.

